# The domesticated brain: genetics of brain mass and brain structure in an avian species

**DOI:** 10.1038/srep34031

**Published:** 2016-09-30

**Authors:** R. Henriksen, M. Johnsson, L. Andersson, P. Jensen, D. Wright

**Affiliations:** 1 AVIAN Behavioural Genomics and Physiology Group, IFM Biology, Linköping University, Linköping 58183, Sweden; 2 Dept of Medical Biochemistry and Microbiology, Uppsala University, BMC, Husargatan 3, Uppsala 75123, Sweden

## Abstract

As brain size usually increases with body size it has been assumed that the two are tightly constrained and evolutionary studies have therefore often been based on relative brain size (i.e. brain size proportional to body size) rather than absolute brain size. The process of domestication offers an excellent opportunity to disentangle the linkage between body and brain mass due to the extreme selection for increased body mass that has occurred. By breeding an intercross between domestic chicken and their wild progenitor, we address this relationship by simultaneously mapping the genes that control inter-population variation in brain mass and body mass. Loci controlling variation in brain mass and body mass have separate genetic architectures and are therefore not directly constrained. Genetic mapping of brain regions indicates that domestication has led to a larger body mass and to a lesser extent a larger absolute brain mass in chickens, mainly due to enlargement of the cerebellum. Domestication has traditionally been linked to brain mass regression, based on measurements of relative brain mass, which confounds the large body mass augmentation due to domestication. Our results refute this concept in the chicken.

Brain size variation across vertebrate species continues to fascinate evolutionary biologists, due in part to the cognitive and behavioral phenotypes it is thought to underlie. Such research is not limited to extant species, with the relationship between brain size and the fossil record capable of identifying the origins and development of avian species, and giving clues as to the evolutionary development of flight^1^,^2^. Most studies on brain size differences suggest some type of trade-off between the costs of developing and maintaining energetically expensive brain tissue and certain physiological variables (such as body size^3^, metabolic rate^4^ and development time^5^) or lifestyle variables (e.g. foraging ecology^6^ and social environment^7^). One physiological variable that correlates notably with brain size is body size^3^. As brain size usually increases as body size increases^8^ it has been assumed that the two are tightly constrained during developmental growth^9^. Researchers have therefore often relied on relative rather than absolute brain size in correlative studies. The relationship between body size and brain size is, however, poorly understood and the use of allometry in brain size evolution studies has been criticized^10^,^11^,^12^. Understanding the genetics of brain size evolution is extremely pertinent to determine the relationship between brain size and body size. Most importantly, to what degree is there overlap (and potential pleiotropy) between the genes responsible for both. To date, studies on the genetic relationship between brain and body size are mostly limited to phylogenetic comparisons and measures of selection, and have failed to identify the overlap of the genetic architecture between these traits, especially using a within-species approach. The analysis of the rates of evolution in a between-species analysis of cichlids indicates that brain size and body size can have distinct rates of evolution^13^. Similarly, selection on body mass can potentially drive reduced relative brain mass^14^, whilst different orders of animals may have different brain-body mass variation, which is driven primarily by variability in body mass^15^. Genetic correlations between brain and body size in stickleback (*Gasterosteus aculeatus*) showed a positive correlation between these two traits^16^, but also a large standard error to this estimate (with the correlation being between 12–96%). In the principle study to actually assess the genetic architecture of quantitative variation in brain size, a quantitative trait loci (QTL) study in inbred mice strains identified genomic regions associated with overall brain mass^17^, though was primarily focussed on identifying QTL for separate brain sub-regions. In this study, however, the genetic architecture for body mass was almost entirely unresolved (identifying only one suggestive locus), making it impossible to assess any potential overlap between brain and body mass loci. Therefore, little is known regarding the combined genetic architectures for brain mass and body mass, and studies that comprehensively examine the genetic architecture of both brain mass and body mass in an intra-species specific manner are lacking.

Most studies examining the genetics of brain and body size in avian species have focussed on the genes underpinning specific brain regions. Genes relating to variation in the brain structures governing song learning^18^,^19^,^20^, and the genetic programs involved in determining the basic architecture of the telencephalon^21^,^22^,^23^, have been examined in particular. However, the genetic basis of overall brain composition differences have yet to be fully investigated in birds, particularly in regards to the genes responsible for quantitative variation in these tissues. The degree to which different brain regions can develop independent of one another is highly debated. According to the ‘mosaic evolution’ hypothesis individual brain regions can develop and grow independently in size^24^ while the ‘concerted evolution’ hypothesis argues that different brain regions have been limited by developmental constraints and that brain size alters predominantly as a whole^5^. In the case of the latter hypothesis therefore individual regions cannot change in size independently. To date, only two studies have attempted to identify gene regions that affect variation in brain substructure size within species. The previously mentioned QTL study in inbred mice strains^17^, and a Genome Wide Association Study (GWAS) study by Hibar *et al*.^25^ that identified eight loci affecting putamen and caudate nucleus volumes in humans.

Brain size differs substantially within species^26^. Since evolution operates through intraspecific variation, within-species differences can help disentangle the relationship between brain and body size and the degree to which different brain structures are constrained developmentally. The process of domestication is especially interesting because of the huge differences in both brain and particularly body size in domesticated animals as compared to their wild progenitors^27^,^28^, and the reduction in relative brain size. This generates a useful model for assessing the effects of variation in brain and body size, how body size is constrained by brain size and vice versa. A classical effect of domestication is reduced relative brain size^29^, with this believed to reflect the reduced functional needs of domesticated animal’s brains^30^. Studies have reported smaller brain size in domesticated mammalian (sheep^31^, pig^32^,^33^, and mink^34^), and avian species (turkeys^35^, chickens^36^, pigeons^29^, and ducks^37^), as compared to their wild progenitors. However, the methods used in virtually all studies on brain size reduction in domesticated animals confound the effect of brain reduction with the often very large body size augmentation that has occurred during domestication. The effect of reduced relative brain size with directional selection is not limited to domestication, with studies on fish showing that reduced relative brain mass can be potentially driven by increased selection for body mass^14^. Thus results from the genetic dissection of domestication may be pertinent in a wider perspective.

Genomic sequencing of the wild progenitor of all domesticated chickens, the Red Junglefowl (RJF) *Gallus gallus*, as well as different domestic chicken breeds has enabled the identification of selective sweeps – gene regions that have been strongly selected upon during domestication^38^. Although the domestic chicken and RJF differ both physiologically and behaviourally they still belong to the same species and can interbreed. This allows us to perform a similar QTL analysis as that by Hager and colleagues in mice^17^ but with the added advantage that large differences in the domestic chicken and RJF brain and body mass and genome provides us with an *a priori* hypotheses regarding brain and body phenotype (mass) and which genes might be involved (underlying selective sweep-regions).

In this study we use a domestic (White Leghorn chicken, *Gallus gallus domesticus*) x wild (RJF) advanced intercross to fine-map quantitative trait loci (QTL) pertaining to brain mass and body mass differences between wild and domestic populations, as well as ontogenetic analyses of brain and body mass development in wild and domestic birds. This intercross allows us to separately map both brain mass and body mass QTL and determine the genetic architecture of both traits. Brains from the majority of this intercross population were subdivided into 4 brain regions and weighed. To assess potential differences in cerebrotype, each brain region was mapped both as a whole-weight measure and in terms of the relative proportion of total brain mass accounted for by each region (termed ‘relative QTL’), i.e. by dividing the weight of each region by the total brain mass. It is important to note that the QTL mapping procedure only detects loci that differ between the parental populations, i.e. those loci that are distinct between wild and domestic birds. Loci that contain polymorphisms that both populations have in common will not be detected. In this way, we can therefore specifically detect those loci that control brain mass and body mass that have been selected upon by domestication. However, the general loci that both domestic chickens and Red Junglefowl share will be undetected, and therefore missed. Ontogenetic brain and body mass development analyses were performed using populations of wild and domestic birds. These allow the developmental growth trajectories of both brain mass and body mass to be compared between wild and domestic birds. If brain mass is indeed inextricably linked with body mass, the growth trajectories of both brain mass and body mass should mirror one another, in both wild and domestic populations. This enables a separate analysis of the relationship between brain and body mass in an inter-population context. To our knowledge, this is the first study to actually identify multiple genomic regions underpinning the mass differences in both whole brain and brain region mass, whilst simultaneously mapping body mass, not only between a wild and domesticated population but also in vertebrates in general. This approach separates and decouples the detected brain loci at a genetic level from those loci affecting only body mass.

## Methods

### Chicken study population and cross design

The intercross population is an eighth generation intercross between a population of RJF, derived originally from Thailand^39^,^40^ and a line of selected White Leghorn (WL) chickens, with a total of 470 adult F_8_ individuals used in this study. Females were assayed for brooding prior to culling. In addition a total of 61 RJF and 65 WL chickens were reared (in conditions identical to the intercross birds) to assess brain development and growth differences from ages one, two, four, ten and fifteen weeks and adult, between RJF and WL. Eleven broiler birds were additionally dissected at two weeks of age, as a comparison for the other major domesticated strain. Volumetric measurements were taken on the brains of two adult male RJF and 2 adult male WL individuals. The study was approved by the local Ethical Committee of the Swedish National Board for Laboratory Animals. All methods were performed in accordance with the relevant guidelines and regulations.

### Phenotyping

#### Brain measurements and dissection

Immediately after culling, the brains were removed from the birds and the whole brain weighed, after which they were dissected into four pieces for 315 individuals and nine pieces for 129 individuals. Total brain mass values were available for 439 of these individuals. Four piece dissections involved dividing the brain into the cerebral hemispheres, optic tectum, cerebellum and a brainstem region (which included thalamus, the rest of the midbrain and the hindbrain; for more information on the dissected brain regions see Supplementary Figure 1 and the Supplementary methods). Nine-piece dissections were only used for QTL analysis of body mass and whole brain mass, with these dissections being used for a different study (ref. 41 – with the brainstem region further subdivided into the optic chiasma, thalamus, hypothalamus, medulla oblongata, pons and nucleus tractus solitarii). Each of the four separate brain regions were weighed to the nearest 0.001 g. To ascertain whether a heavier brain also equates to a larger brain, brain mass was compared with volume in a subset of RJF and WL birds. The relative brain region mass was calculated by dividing the mass for each specific region by the total brain mass. Details of volumetric measures are given in the Supplementary methods section as is the technique for the assessment of brooding. Brain volume was found to scale linearly with mass (see Supplementary Figure 2A). In all four brain regions, regions with a larger mass had a greater volume, with brain mass explaining 98–100% of the variation when regressed on to brain volume (Supplementary Figure 2B).

#### Genotyping, QTL and mapping

All individuals were genotyped for 652 SNP markers, with QTL analysis performed using R/Qtl^42^ for both standard interval mapping and epistatic analyses. See Supplementary methods for full details of the mapping and models used.

## Results

### Total brain mass is larger in domestic birds with a different growth trajectory relative to body mass in comparison to wild birds

**Figure 1:**
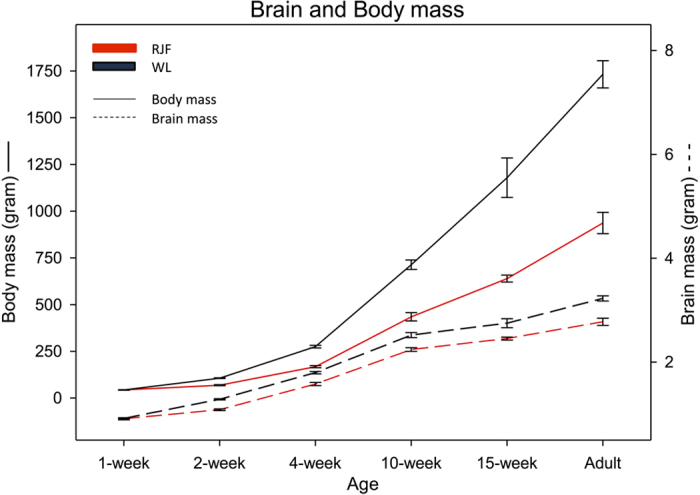
Changes in body mass (solid lines: mean ± SE) and brain mass (dotted lines: mean ± SE) in White leghorn (black lines) and Red Junglefowls (red lines) from 1-week of age until adulthood.

Absolute brain mass is larger in domestic (WL) chickens relative to wild RJF (Fig. 1), with a consistent difference of approximately ~0.2 g up to 5 weeks of age, with this increasing to ~0.4 g at sexual maturity (adult RJF brain mass (mean ± S.D.) 2.78 g ± 0.22, adult domestic brain mass 3.22 g ± 0.20). In the case of both domestic chickens and RJF brain growth is linear up to adulthood. In contrast, body mass differences diverge rapidly after 4 weeks of age between domestic chickens and RJF, with domestic chickens showing a sharp exponential increase in mass, and at sexual maturity they weigh almost twice as much as RJF (Fig. 1). Thus whilst domestic chickens exhibit the classical effects of domestication (reduced relative brain mass, massively increased body mass), the differences between brain growth and body growth trajectories between these two populations suggests that different genetic systems are at play governing these traits. If this is the case, then the genetic architecture of brain mass and body mass in the intercross population should be non-overlapping. Additionally, the brain composition of wild birds and domestic birds was also found to differ, with domestic birds possessing a larger cerebellum and cerebral hemispheres and smaller optic tecta and brainstem with these differences being largely consistent throughout post-hatch growth (Supplementary Figure 3). Therefore, non-overlapping architecture for different brain sub-regions in the intercross would also favour the mosaic brain theory over the concerted brain hypothesis.

### The detected architectures for inter-population brain mass and body mass appear separate

**Figure 2:**
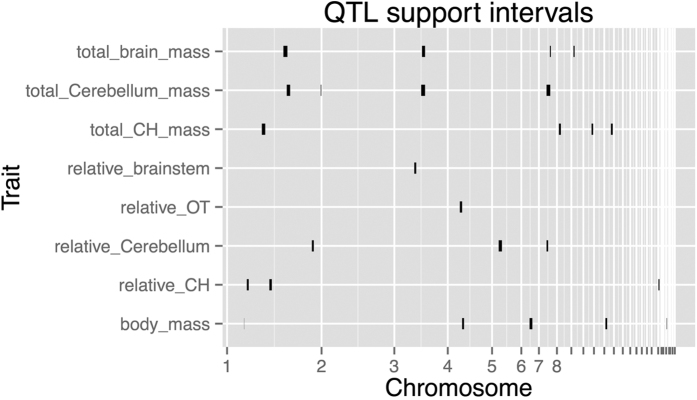
QTL supports intervals (as determined by the 1.8 LOD drop method) in the genome separating out loci for relative and total brain mass and body mass. The x-axis represents the chromosome containing the QTL, whilst the size of the each bar represents the total QTL region size (i.e. the 95% C.I. of the QTL location).

**Figure 3:**
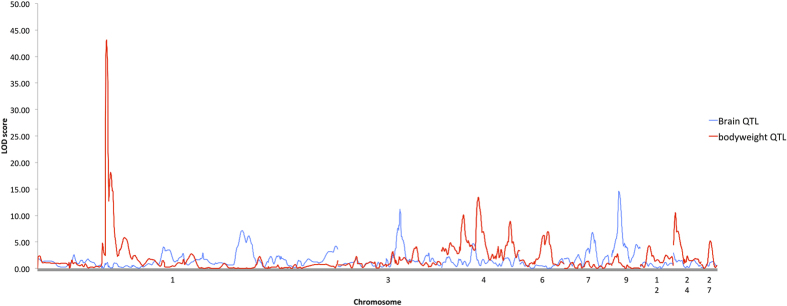
Comparison of LOD graphs for total brain mass QTL and body mass QTL.

A total of 20 QTL relating to inter-population brain mass and relative brain mass variation were identified (Table 1 and Supplementary Table 2). One of the first things of note is that the QTL for total brain mass, relative brain region mass and absolute brain region mass are entirely separate from the QTL for body mass. There is no overlap for brain and body mass loci, indicating that the genes selected by domestication for body mass and brain region mass are at least partially separate, i.e. it is possible to select for increased brain growth without increased body mass and vice versa during domestication (see Figs 2 and 3). Although body mass was not used as a covariate for the brain mass QTL analysis presented above (due to the possibility that including it could mask QTL that overlap body mass by already factoring body mass out the model), its inclusion led to no changes in the detected QTL position, and in fact strengthened the QTL in some cases, further highlighting the separate nature of the two architectures. A general caveat with QTL analysis is that the genes controlling brain mass and body mass that are shared between domestic and wild populations (i.e. intra-population variation) will not be detected as QTL in this analysis. However, the phenotypic correlation between brain and body mass in this cross is relatively low and undetected loci causing intra-population variation that also overlapped would increase this correlation. Although significant, sex and body mass together account for only 18% of the total variation in brain mass, with sex alone accounting for 16% of the variation. Similarly, when a linear model fitting all the detected QTL as well as body mass was regressed onto brain mass, body mass still only accounted for 2% of the variation in brain mass variation, whilst the detected genetic loci accounted for 22% of the brain mass variation. A further caveat is that genes with effects too small to be detected may yet be pleiotropic between brain and body mass, though given the lack of any phenotypic correlations as noted above, this seems less probable. In the case of the genetic architecture for body mass, the detected QTL account for over 36% of the total variation for body mass (excluding sex differences), whilst the total GLM that included all body mass QTL and other covariates explained 84% of the variation for body mass. Therefore, relatively little unexplained variation is present in the model for body mass. A significant sex interaction can indicate that a QTL has greater effect on one sex as compared to the other. In this case, very few sex interaction effects were seen for cerebellum and total brain mass traits, with the only interactions being on chromosomes 3 (total brain mass) and 5 (relative cerebellum mass), with none being found for any other traits (see Supplementary Table 3).

### Domestic genotypes cause larger brains and larger cerebella than wild genotypes

In the case of the cerebellum and total brain mass QTL, the observed allelic effect is in accordance with the phenotypic differences seen in the wild and domestic breeds (see Supplementary Figures 3 and 4). Domestic alleles correlate with an increase in cerebellum mass and total brain mass in 6 out of 7 of the cerebellum QTL and 3 out of 4 of the brain mass QTL identified, i.e. the QTL are not transgressive (see the additive and dominance QTL effect sizes in Table 1), meaning that the alleles correlating with an increase in the phenotype come from the parental strain possessing the larger phenotype. In combination with the observation that brain mass has a separate genetic architecture to body mass in this cross, this indicates that once the genes underpinning brain mass and body mass are de-coupled, domestic chickens have larger, not smaller, brains as compared to their wild counterparts. Further to this, the cerebellum is also larger in mass in the domestic chickens (both totally and as a proportion of total brain mass), with the genetic control of this trait separate from overall body mass. There is a strong overlap between the observed genetic architectures for the cerebellum and total brain mass, with three of the four brain mass QTL overlapping total cerebellar mass QTL (on chromosomes 1, 3, and 7, see Table 1).

### Brain regions each have their own unique genetic architecture

The different brain regions have separate genetic architectures from one another for both total and relative mass, as determined by the loci detected. Although in the case of the optic tectum and brainstem regions very few QTL were identified (only two loci in total). For both cerebral hemispheres and the cerebellum multiple QTL were identified (18 in total), explaining a relatively large amount of the variation in the cross (26% of total variation for relative cerebral hemispheres mass, 26% of total variation for total cerebellum mass, 22% of the variation for total brain mass). Even with these more complete genetic architectures, there was nevertheless no overlap between different brain regions QTL with one another, indicating that, at least partially, this supports the mosaic theory of brain evolution^24^.

### Brain mass is selected for during domestication

Selective sweeps have been putatively identified in the chicken genome, representing regions that have reduced heterozygosity during the periods of selection that occurred during domestication^38^. By using a clustering analysis based on the number of sweeps detected in the genome, the confidence intervals of the brain QTL and the number of overlaps between brain QTL and selective sweep loci, we found that the QTL relating to cerebellum mass (7 loci in total, marked in bold in Table 1) contained a significant enrichment of selective sweeps (P = 0.04, using the clustering test as described in the Supplementary methods), as did the regions relating to total brain mass (4 loci overlapped, P = 0.01, clustering test). These results indicate that loci affecting cerebellum and total brain mass may have been directly selected upon during domestication, or that these loci are closely linked with other genes targeted by domestication selection. Given this significant overlap between selective sweeps and QTL regions, genes contained within these sweeps are good candidates for the genes causal to the QTL (often referred to as Quantitative Trait Genes). Twelve genes are present in the selective sweeps within total brain and cerebellar QTL (see Table 1). Four of these (*FGF3, FGF9, BIN1, SHANK1*) have previous associations with neuronal conditions or neurogenesis (see discussion).

### Brooding behaviour correlates with relative cerebellum mass

Given the strong selection on the mass of the cerebellum in domestic chickens, we correlated brooding behaviour with relative cerebellum mass. We found a strong negative relationship between brooding behaviour and relative cerebellum mass (GLM, n = 123, F = −2.6, P = 0.009), indicating that birds with a larger cerebellum and thereby a more domesticated brain cerebrotype displayed less brooding behaviour, characteristic of domestic chickens. Body mass was included in the model as a covariate, so these results are not due to overall body mass effects. Four QTL were detected for brooding behaviour (see Supplementary Table 2). Although none overlapped total brain mass QTL, the confidence interval of one (on chromosome 9) was within 32 cM of a total brain mass QTL, whilst another (on chromosome 4) was within 38 cM of a QTL for relative optic tectum mass. In the case of the latter this distance corresponds to around 200 kb, whilst in the case of the latter it is around 10 Mb. No correlations were found between other brain regions and brooding behaviour.

### RJF and WL fat/lean/bone body tissue proportions

The body composition, and in particular the lean mass to fat mass percentage, could potentially be important in explaining intra-species brain mass differences, especially if lean mass requires a proportional increase in neurons. If domestic birds have a far greater percentage of fat mass, then departures from the usual brain allometry may be due to a large brain mass not being required for the extra fat tissue they possess. Using data from a previous study by Rubin and colleagues^43^ that measured overall fat, lean and bone mass in both wild and domestic chickens using Dual X-ray Absorbancy (DXA) techniques it is possible to calculate the relative proportions of these tissue types (see Supplementary Table 4). Although domestic females have a higher percentage of body fat (65% lean mass in WL females to 84% lean mass in RJF females), the domestic males (that show the largest decrease in relative brain mass) actually possess a far lower percentage body fat than their wild counterparts, with a correspondingly higher proportion of lean muscle mass (86% lean mass in WL males to 77% lean mass in RJF males).

## Discussion

Domesticated chickens (WL) have a larger brain mass and body mass than their wild progenitor, but whereas body mass has increased by ~85% during domestication, brain mass has only increased by ~15%. This indicates that brain mass has been altered less by selection during domestication than body mass and that in chickens reduced relative brain mass during domestication has mainly been caused by an increase in body mass. By separating the linkage between loci affecting inter-population variation in brain mass and body mass in an advanced intercross we demonstrate that domestication selection has acted on apparently separate loci to increase brain mass and body mass in domesticated individuals. Therefore selection on body mass is not limited by brain mass and vice versa in the chicken, with domestication leading to (at least partially) separate genetic architectures for these traits. It is important to note that brain and body mass architectures that are common to both wild and domestic populations will not be detected with this analysis. This means we cannot infer that no loci affecting brain mass and body mass are pleiotropic or overlap, only that those that are due to domestication selection do not. Similarly, with any QTL study there is always the possibility that loci with effect sizes too small to detect exist and do overlap and exhibit pleiotropy. However, the relatively small percentage of variation of brain mass explained by body mass suggests that these within-population loci are of smaller effect than the between-population loci. In the combined model of QTL and body mass covariates affecting brain mass, body mass explains only 2% of brain mass variation whilst the QTL explains around 22%, and sex explains 4% of the variation. Likewise, if numerous undetected, small-effect loci were also pleiotropic we would still expect the brain and body mass correlation to be higher. Our study is the first to provide evidence for a relatively distinct genetic architecture for body mass and brain mass in an avian species and also to demonstrate that separate loci underlie the mass of the different brain regions, thereby showing that the mass of an individual brain region can be selected upon without being strictly constrained by the mass of the other brain regions, as predicted by the mosaic brain evolution hypothesis. Support for mosaic brain evolution has also been reported in mammals^17^ and fish^16^ and together with our findings in birds suggest that mosaic brain evolution is possible across vertebrate species.

The nature of the body and brain mass increases during domestication is of relevance when considering allometric scaling and the relationship between brain mass and body mass. It has been proposed that the developmental constraints limiting brain and body mass are related to the overall musculature (see the trophic theory of neural connections^44^, amongst others), with increased neural circuitry required for an increase in muscular anatomy. A possibility therefore is that domestic birds have a lower relative brain mass due to increased fat reserves making up a larger percentage of their overall body mass. The majority of the mass gain in domestics would then be due to increased fat deposits and thus not require any increase in brain mass. However, the calculations we performed using the data from a previous study by Rubin and colleagues^43^ indicates that this does not appear to be a limiting factor in this instance, with male WL birds having a higher lean body mass percentage than their RJF counterparts. Similarly, we have also shown that in chickens an increase in brain mass also correlates with an increase in brain volume. Similar strong correlations between brain mass and brain volume have recently been shown in stickleback fish^16^. This suggests that the density of brain tissue is constant, however it remains an open and intriguing question whether the increased brain mass induced by domestication relates to the incorporation of more neurons, or alternatively non-neuronal cells such as blood cells or glial cells, connections and the like.

Although the brain and the four different brain regions grow continuously until adulthood in both RJF and WL, the relative mass of each brain region changes during posthatch development and domestic and wild birds differ in the relative mass of certain brain regions. These differences in cerebrotype between RJF and WL, caused by selection during domestication, must therefore occur initially during prehatch development or during the first week posthatch. Our findings suggest that selection during domestication in chickens has been stronger on the cerebral hemispheres and cerebellum than on the brainstem region and optic tectum. This is further supported by the enrichment of selective sweeps (gene regions that have been strongly selected upon during chicken domestication), which are present in the QTL regions for both cerebellum and overall brain mass. Increased relative cerebellum mass, compared to their wild counterparts, is seen in most studies on domesticated birds, including domesticated geese^45^, turkeys^35^ and pigeons^46^ (but see also^29^ for opposite results), but not in ducks^37^, suggesting that relative enlargement of the cerebellum may have played an important role during domestication in several birds species.

We find that brooding behaviour is inversely correlated with the relative mass of the cerebellum. These findings suggest that the cerebellum could help govern this behaviour, and therefore provide a link between domestication effects on brain composition (an enlarged cerebellum) and domestication effects on behaviour (reduced brooding behaviour). Relative enlargement of the cerebellum and cerebral hemispheres could potentially have been important for the chicken to adapt to several aspects of the domesticated environment. In birds increased cerebral hemispheres mass has been linked with increased social complexity^47^ and the cerebellum has been linked with foraging strategy^48^. Wild RJF live in small group sizes of around 4–10 individuals^49^, whereas domestic chickens are kept in far larger groups. Although these theories are tempting to extrapolate upon, care must be taken in their interpretation given the purely correlative data.

The ultimate goal of a QTL study is often to identify the genes themselves that underlie the trait in question. In standard (F_2_ or similar) QTL analyses, the confidence intervals of detected QTL are so large (typically in the region of 20–30 megabases) that identifying putative candidate genes is virtually impossible. However, in the study presented here the combination of the advanced intercross and the overlap with selective sweeps for domestication yield a number of candidate genes for brain growth. In regards to the QTL for cerebellum mass and total brain mass, 12 genes in total are present within sweeps in these regions. Of these, four have prior associations with neural conditions and neuronal generation, reinforcing them as excellent candidate genes for increased brain and cerebellum mass. The chromosome 1 sweep at 182.6 Mb, in the middle of the QTL region for total cerebellum mass, contains the gene *FGF9*, which regulates the generation and positioning of Bergmann glial cells in the developing mouse cerebella^50^. *FGF9* also has a crucial role in embryonic neurological development^51^,^52^,^53^,^54^. Similarly the QTL for relative cerebellum mass on chromosome 5 contains 4 sweeps, with three genes present in those sweeps. Once again an *FGF* gene, in this case *FGF3*, is present. Also present in a separate sweep is *SHANK1*, mutations in which are associated with dysfunction of glutamatergic synapses that lead to a variety of neuropsychiatric disorders in humans including autism and schizophrenia^55^. The sweep located at 25.42 Mb on chromosome 7 that overlaps with the total brain mass QTL is located within 100 kb of the gene *BIN1* that has effects on working memory, hippocampal volume and functional connectivity^56^. The QTL for total cerebellum mass on chromosome 1 at 2204 cM has an interval of 1.1 Mb and is therefore sufficiently highly resolved to also address the genes contained within for potential functionality, containing as it does 15 reference sequence genes. Of these, a number of highly interesting genes are identified for further investigation. *MAP6* is in the centre of the QTL confidence interval and mediates neuronal connectivity for axonal growth^57^. It has been linked with synaptic plasticity anomalies and has associations with schizophrenia through neuronal transport defects^58^ and cognitive impairment^59^. *CCKBR* is linked with posttraumatic stress disorder and synaptic plasticity^60^ and *PLEKHB1* is associated with Attention Deficit Hyperactivity Disorder^61^.

In summary, our findings refute the common notion that domestication has led to the regression of brain mass in the domestic chicken. We show that domestication has led to the enlargement of the cerebellum and cerebral hemispheres, and have identified genomic regions containing selective sweeps that in turn give us a number of candidate genes that have putative effects on variation in total brain and cerebella growth. This demonstrates that the RJF and the domestic chicken provide an interesting animal model for studying brain mass evolution during domestication, and also a general model for studying evolution in brain mass and brain composition.

## Additional Information

**How to cite this article**: Henriksen, R. *et al*. The domesticated brain: genetics of brain mass and brain structure in an avian species. *Sci. Rep.* **6**, 34031; doi: 10.1038/srep34031 (2016).

## Supplementary Material

Supplementary Information

Supplementary Dataset

## Figures and Tables

**Table 1:** QTL information for body mass, total brain mass and cerebellum QTL.

Trait	chr	pos	LOD	r-sq	add +/− s.e	dom +/− s.e.	lower CI	upper CI	selective sweeps present
body mass (212 days)	1	510	43.5	21.5	235 +/− 28	−20 +/−38	507	516	
total brain mass	1	1516	5.3	3.3	0.06 +/− 0.02	−0.06 +/−0.04	1494	1583	119.46 LR (*SPAC17A2*), 127.88 LR (*ARHGAP6*)
total Cerebellum mass	1	1593	13.5	9.9	0.012 +/−0.005	−0.023 +/−0.007	1586	1598	127.88 LR
relative Cerebellum	1	1945	4.8	4.8	0.003 +/−0.001	0.005 +/−0.002	1931	1956	179.66 LR (*UBL3*), 182.6 LR (*FGF9*)
total Cerebellum mass	1	2204	9.3	6.6	0.0004 +/−0.004	−0.004 +/−0.005	2196	2224	*MAP6, CCKBR, PLEKHB1*
total Cerebellum mass	3	448	8.5	6	0.005 +/−0.005	0.022 +/−0.007	442	458	62,62 LR (*KNF217*), 64.04 LR (*SERINC1*)
total brain mass	3	448	8.7	5.6	−0.12 +/−0.07	0.25 +/−0.09	444	454	62,62 LR (*KNF217*), 64.04 LR (*SERINC1*)
body mass (212 days)	4	265	8.6	4.7	8.1 +/−9.8	19.8 +/−12.3	254	274	
relative Cerebellum	5	124	4.8	4.8	0.002 +/−0.004	−0.03 +/−0.006	106	144	18.8 LR (*FGF3*), 19.4 LR (*SHANK1*), 20.2 LR (*CAT*), 20.5 LR (*CD44*)
body mass (212 days)	6	207	5	1.9	26 +/−8	30 +/−11	195	214	
relative Cerebellum	7	159	7.4	7.6	0.005 +/−0.002	0.004 +/−0.002	150	171	
total Cerebellum mass	7	174	5.9	4.1	0.019 +/−0.005	0.003 +/−0.005	150	176	23.04 AD (*TANK1*)
total brain mass	7	200	6.6	4.2	0.02 +/−0.018	−0.04 +/−0.03	190	212	23.04 AD (*TANK1*), 23.1 AD, 25.42 LR (*BIN1*), 25.9 LR
total brain mass	9	51.1	13.6	8.9	0.018 +/−0.017	−0.04 +/−0.02	48	60	
body mass (212 days)	12	64	4.4	1.7	35 +/−10	20 +/−14	45	79	
body mass (212 days)	24	14	9.5	5	0.8 +/−9.1	38 +/−11	6	18	
body mass (212 days)	27	68	4.2	1.6	38 +/−11	−1.8 +/−9.8	56	80	

Table contains QTL locations (both the chromosome and the position in centiMorgans), % variance explained by each QTL (r-squared), additive and dominance effect sizes (positive values for additive values indicate a larger QTL effect size in domestic genotype birds, negative a larger value in wild genotype birds). The lower and upper bounds of the 95% confidence interval (C.I) are noted. The total QTL region is therefore the region bounded between these two limits. Locations of selective sweeps are also provided, with AD indicating the sweep is present in both Broiler and Layer birds, and LR indicating the sweep is specific to Layer birds. For sweeps present in cerebellum and total brain mass QTL any genes present within these sweeps are also provided after the sweep location. Cerebellum QTL are marked in bold.

## References

[b1] Balanoff A. M., Bever G. S., Rowe T. B. & Norell M. A. Evolutionary origins of the avian brain. Nature 501, 93–96 (2013). 23903660 10.1038/nature12424

[b2] Balanoff A. M. . Best practices for digitally constructing endocranial casts: examples from birds and their dinosaurian relatives. Journal of Anatomy (2015). 10.1111/joa.12378 PMC4948053 26403623

[b3] Harvey P. & Pagel M. The allometric approach to species differences in brain size. Human Evolution 3, 461–472 (1988).

[b4] Isler K. & Van Schaik C. P. Metabolic costs of brain size evolution. Biology Letters 2, 557–560 (2006). 17148287 10.1098/rsbl.2006.0538 PMC1834002

[b5] Finlay B. L. & Darlington R. B. Linked regularities in the development and evolution of mammalian brains. Science 268, 1578–1584 (1995). 7777856 10.1126/science.7777856

[b6] Hutcheon J. M., Kirsch J. A. & Garland T. Jr. A comparative analysis of brain size in relation to foraging ecology and phylogeny in the Chiroptera. Brain, Behavior and Evolution 60, 165–180 (2002). 10.1159/000065938 12417821

[b7] Dunbar R. I. & Shultz S. Evolution in the social brain. Science 317, 1344–1347 (2007). 17823343 10.1126/science.1145463

[b8] Barton R. A. Primate brain evolution: integrating comparative, neurophysiological, and ethological data. Evolutionary Anthropology: Issues, News, and Reviews 15, 224–236 (2006).

[b9] Finlay B. L., Darlington R. B. & Nicastro N. Developmental structure in brain evolution. Behavioral and Brain Sciences 24, 263–278 (2001). 11530543

[b10] Willemet R. Reconsidering the evolution of brain, cognition, and behavior in birds and mammals. Frontiers in Psychology 4 (2013). 10.3389/fpsyg.2013.00396 PMC3696912 23847570

[b11] Schoenemann P. T. Evolution of the size and functional areas of the human brain. Annu. Rev. Anthropol. 35, 379–406 (2006).

[b12] Montgomery S., Capellini I., Barton R. & Mundy N. Reconstructing the ups and downs of primate brain evolution: implications for adaptive hypotheses and Homo floresiensis. BMC Biology 8, 9 (2010). 20105283 10.1186/1741-7007-8-9 PMC2825212

[b13] Gonzalez-Voyer A., Winberg S. & Kolm N. Distinct evolutionary patterns of brain and body size during adaptive radiation. Evolution 63, 2266–2274, 10.1111/j.1558-5646.2009.00705.x (2009). 19473380

[b14] Fitzpatrick J. L. . Sexual selection uncouples the evolution of brain and body size in pinnipeds. Journal of Evolutionary Biology 25, 1321–1330, 10.1111/j.1420-9101.2012.02520.x (2012). 22530668

[b15] Smaers J. B., Dechmann D. K. N., Goswami A., Soligo C. & Safi K. Comparative analyses of evolutionary rates reveal different pathways to encephalization in bats, carnivorans, and primates. Proceedings of the National Academy of Sciences of the USA 109, 18006–18011, 10.1073/pnas.1212181109 (2012). 23071335 PMC3497830

[b16] Noreikiene K. . In *Proceedings of the Royal Society London B*. 20151008 (The Royal Society).

[b17] Hager R., Lu L., Rosen G. D. & Williams R. W. Genetic architecture supports mosaic brain evolution and independent brain–body size regulation. Nature Communications 3, 1079 (2012). 10.1038/ncomms2086 PMC4267555 23011133

[b18] Jarvis E. D. . Behaviourally driven gene expression reveals song nuclei in hummingbird brain. Nature 406, 628–632 (2000). 10949303 10.1038/35020570 PMC2531203

[b19] Brenowitz E. A. Plasticity of the adult avian song control system. Annals of the New York Academy of Sciences 1016, 560–585 (2004). 15313795 10.1196/annals.1298.006

[b20] Brenowitz E. A. Comparative approaches to the avian song system. Journal of neurobiology 33, 517–531 (1997). 9369457 10.1002/(sici)1097-4695(19971105)33:5<517::aid-neu3>3.0.co;2-7

[b21] Chen C. C., Winkler C. M., Pfenning A. R. & Jarvis E. D. Molecular profiling of the developing avian telencephalon: regional timing and brain subdivision continuities. Journal of Comparative Neurology 521, 3666–3701 (2013). 23818174 10.1002/cne.23406 PMC3863995

[b22] Dugas-Ford J., Rowell J. J. & Ragsdale C. W. Cell-type homologies and the origins of the neocortex. Proceedings of the National Academy of Sciences 109, 16974–16979 (2012). 10.1073/pnas.1204773109 PMC3479531 23027930

[b23] Jarvis E. D. . Global view of the functional molecular organization of the avian cerebrum: mirror images and functional columns. Journal of Comparative Neurology 521, 3614–3665 (2013). 23818122 10.1002/cne.23404 PMC4145244

[b24] Barton R. A. & Harvey P. H. Mosaic evolution of brain structure in mammals. Nature 405, 1055–1058 (2000). 10890446 10.1038/35016580

[b25] Hibar D. P. . Common genetic variants influence human subcortical brain structures. Nature 520, 224–229, 10.1038/nature14101 (2015). 25607358 PMC4393366

[b26] Gonda A., Herczeg G. & Merilä J. Evolutionary ecology of intraspecific brain size variation: a review. Ecology and Evolution 3, 2751–2764 (2013). 24567837 10.1002/ece3.627 PMC3930043

[b27] Jensen P. & Andersson L. Genomics Meets Ethology: A New Route to Understanding Domestication, Behavior, and Sustainability in Animal Breeding. AMBIO: A Journal of the Human Environment 34, 320–324 (2005). 10.1639/0044-7447(2005)034[0320:gmeanr]2.0.co;2 16092263

[b28] Jensen P. & Wright D. In Genetics and behavior of domestic animals (eds Grandin T. & Deesing M. J.) 41–80 (Academic Press, 2014).

[b29] Rehkämper G., Frahm H. D. & Cnotka J. Mosaic evolution and adaptive brain component alteration under domestication seen on the background of evolutionary theory. Brain, Behavior and Evolution 71, 115–126 (2007). 10.1159/000111458 18032887

[b30] Price E. O. Animal domestication and behaviour. (CABI Publishing, 2002).

[b31] Ebinger P. A cytoarchitectonic volumetric comparison of brains in wild and domestic sheep. Zeitschrift fuer Anatomie und Entwicklungsgeschichte 144, 267–302 (1974). 10.1007/BF00522811 4411618

[b32] Plogmann D. & Kruska D. Volumetric comparison of auditory structures in the brains of European wild boars (*Sus scrofa*) and domestic pigs (*Sus scrofa f. dom.*). Brain, Behavior and Evolution 35, 146–155 (1990). 10.1159/000115863 2375973

[b33] Kruska D. Vergleichend cytoarchitektonische Untersuchungen an Gehirnen von Wild-und Hausschweinen. Zeitschrift für Anatomie und Entwicklungsgeschichte 131, 291–324 (1970). 5471597

[b34] Kruska D. The effect of domestication on brain size and composition in the mink (*Mustela vison*). Journal of Zoology 239, 645–661 (1996).

[b35] Ebinger P. & Röhrs M. Volumetric analysis of brain structures, especially of the visual system in wild and domestic turkeys (*Meleagris gallopavo*). Journal fur Hirnforschung 36, 219–228 (1994). 7615926

[b36] Jackson S. & Diamond J. Metabolic and digestive responses to artificial selection in chickens. Evolution, 1638–1650 (1996). 10.1111/j.1558-5646.1996.tb03936.x 28565716

[b37] Ebinger P. Domestication and plasticity of brain organization in mallards (*Anas platyrhynchos*). Brain, Behavior and Evolution 45, 286–300 (1995). 10.1159/000113557 7620877

[b38] Rubin C.-J. . Whole-genome resequencing reveals loci under selection during chicken domestication. Nature 464, 587–591 (2010). 20220755 10.1038/nature08832

[b39] Schutz K. . QTL analysis of a red junglefowl x White Leghorn intercross reveals trade-off in resource allocation between behavior and production traits. Behavior Genetics 32, 423–433 (2002). 12467340 10.1023/a:1020880211144

[b40] Schutz K. E. . Major growth QTLs in fowl are related to fearful behavior: possible genetic links between fear responses and production traits in a red junglefowl x white leghorn intercross. Behavior Genetics 34, 121–130 (2004). 14739702 10.1023/B:BEGE.0000009481.98336.fc

[b41] Johnsson M., Williams M. J., Jensen P. & Wright D. Genetical Genomics of Behavior: A novel chicken genomic model for anxiety behavior. Genetics 202, 327–340 (2016). 26733665 10.1534/genetics.115.179010 PMC4701096

[b42] Broman K. W., Wu H., Sen S. & Churchill G. A. R/qtl: QTL maping in experimental crosses. Bioinformatics 19, 889–890 (2003). 12724300 10.1093/bioinformatics/btg112

[b43] Rubin C.-J. . Quantitative trait loci for BMD and bone strength in an intercross between domestic and wildtype chickens. Journal of Bone and Mineral Research 22, 375–384 (2007). 17181401 10.1359/jbmr.061203

[b44] Purves D. Body and brain: a trophic theory of neural connections. (Harvard University Press, 1988). 10.1126/science.244.4907.993 17731884

[b45] Ebinger P. & Löhmer R. A volumetric comparison of brains between greylag geese (*Anser anser L.*) and domestic geese. Journal fur Hirnforschung 28, 291–299 (1986). 3668239

[b46] Ebinger P. & Löhmer R. Comparative quantitative investigations on brains of rock doves, domestic and urban pigeons (Columba 1. livia) 1. Journal of Zoological Systematics and Evolutionary Research 22, 136–145 (1984).

[b47] Burish M. J., Kueh H. Y. & Wang S.-H. Brain architecture and social complexity in modern and ancient birds. Brain, Behavior and Evolution 63, 107–124 (2004). 10.1159/000075674 14685004

[b48] Iwaniuk A. N. & Hurd P. L. The evolution of cerebrotypes in birds. Brain, Behavior and Evolution 65, 215–230 (2005). 10.1159/000084313 15761215

[b49] Collias N. E. & Collias E. C. A field study of the red jungle fowl in north-central India. Condor, 360–386 (1967).

[b50] Meier F. . FGF/FGFR2 Signaling Regulates the Generation and Correct Positioning of Bergmann Glia Cells in the Developing Mouse Cerebellum. PLoS One 9, e101124, 10.1371/journal.pone.0101124 (2014). 24983448 PMC4077754

[b51] Guillemot F. & Zimmer C. From Cradle to Grave: The Multiple Roles of Fibroblast Growth Factors in Neural Development. Neuron 71, 574–588, http://dx.doi.org/10.1016/j.neuron.2011.08.002 (2011). 21867876 10.1016/j.neuron.2011.08.002

[b52] Ozawa K., Uruno T., Miyakawa K., Seo M. & Imamura T. Expression of the fibroblast growth factor family and their receptor family genes during mouse brain development. Molecular Brain Research 41, 279–288 (1996). 8883961 10.1016/0169-328x(96)00108-8

[b53] Pankratz M. T. . Directed neural differentiation of human embryonic stem cells via an obligated primitive anterior stage. Stem Cells 25, 1511–1520 (2007). 17332508 10.1634/stemcells.2006-0707 PMC2743478

[b54] Bertrand V., Hudson C., Caillol D., Popovici C. & Lemaire P. Neural tissue in ascidian embryos is induced by FGF9/16/20, acting via a combination of maternal GATA and Ets transcription factors. Cell 115, 615–627 (2003). 14651852 10.1016/s0092-8674(03)00928-0

[b55] Schmeisser M. J. Translational neurobiology in Shank mutant mice - Model systems for neuropsychiatric disorders. Annals of Anatomy - Anatomischer Anzeiger 200, 115–117, http://dx.doi.org/10.1016/j.aanat.2015.03.006 (2015). 25917711 10.1016/j.aanat.2015.03.006

[b56] Zhang X. . Bridging integrator 1 (BIN1) genotype effects on working memory, hippocampal volume, and functional connectivity in young healthy individuals. Neuropsychopharmacology (2015). 10.1038/npp.2015.30 PMC4915264 25630570

[b57] Deloulme J.-C. . Microtubule-associated protein 6 mediates neuronal connectivity through Semaphorin 3E-dependent signalling for axonal growth. Nat Commun 6, 10.1038/ncomms8246 (2015). PMC4468860 26037503

[b58] Daoust A. . Neuronal transport defects of the MAP6 KO mouse – a model of schizophrenia – and alleviation by Epothilone D treatment, as observed using MEMRI. NeuroImage 96, 133–142, http://dx.doi.org/10.1016/j.neuroimage.2014.03.071 (2014). 24704457 10.1016/j.neuroimage.2014.03.071

[b59] Fournet V. . The deletion of STOP/MAP6 protein in mice triggers highly altered mood and impaired cognitive performances. Journal of Neurochemistry 121, 99–114, 10.1111/j.1471-4159.2011.07615.x (2012). 22146001

[b60] Chakraborty N. . Gene and stress history interplay in emergence of PTSD-like features. Behavioural Brain Research 292, 266–277, http://dx.doi.org/10.1016/j.bbr.2015.05.038 (2015). 26025510 10.1016/j.bbr.2015.05.038

[b61] Lesch K. P. . Genome-wide copy number variation analysis in attention-deficit/hyperactivity disorder: association with neuropeptide Y gene dosage in an extended pedigree. Mol Psychiatry 16, 491–503, http://www.nature.com/mp/journal/v16/n5/suppinfo/mp201029s1.html (2011). 20308990 10.1038/mp.2010.29

