# Supplementary material for: The domesticated brain: genetics of brain mass and brain structure in an avian species
Source: Sci Rep. 2016 Sep 30;6:34031. doi: 10.1038/srep34031 (PMC5043184; doi:10.1038/srep34031)
Supplement: Supplementary Information [file srep34031-s1.pdf]

The domesticated brain:
genetics of brain mass and brain structure in an avian species

Henriksen, R.[1], Johnsson, M.[1], Andersson, L.[2], Jensen, P.[1], Wright, D.[1]

[1]AVIAN Behavioural Genomics and Physiology Group, IFM Biology, Linköping University, Linköping 58183, Sweden

[2] Dept of Medical Biochemistry and Microbiology, Uppsala University, BMC, Husargatan 3, Uppsala 75123, Sweden

SUPPLEMENTARY METHODS

Brain regions:

The four regions the brains were divided into are referred to as Cerebral hemisphere, Optic tectum, Brainstem and Cerebellum throughout the study. The cerebral hemisphere region refers to the two cerebral hemispheres, which constitutes the larges part of the avian brain [1]. The optic tectum (sometimes also called optic lobes [2]) is a major part of the midbrain in birds [3,4]. The cerebellum is part of the hindbrain and is located at the back of the scull [1]. The brainstem region of this study includes the remaining part of the brain: including the brainstem-area [5] and thalamus [1]. The brainstem-area includes the midbrain-area (minus optic tectum) and hindbrain-area (minus cerebellum).

Brain Volume Measurements

Volumetric Measurements of Brain Regions

Volumetric brain measurements were taken of four adult male chickens (two Red Junglefowl and two White Leghorn domestic layers). Brains were dissected out from birds, before being weighed in the same manner as stated previously. Brain volumes were calculated using the method detailed in [6] using a principal based on the changing mass measurements of the brains when suspended in water as compared to their standard weight. This method is used due to the increased accuracy as compared to more typical water displacement methods. A Pearson correlation was used to test the correlation between total brain mass and total brain volume using the R- statistical software package [7].

Genotyping, QTL and mapping

DNA preparation was performed by Agowa GmbH (Berlin, Germany), using a standard salt extraction technique [8]. A total of 652 SNP markers were used to generate a map of length ~92675cM, with an average marker spacing of ~16cM. SNPS were chosen based on a previously obtained panel of 10000 SNPs that had been run on the parental birds. Additional details of marker generation, map generation and the like can be found in [9]. QTL analysis was performed using R/Qtl [10] for both standard interval mapping and epistatic analyses. Interval mapping was performed using additive and additive+dominance models. Map generation and permutation threshold measures were performed using the F_8 dataset, to account for the map expansion from the F_2 to the F_8. In the body mass QTL analysis batch and sex were always included in the model as fixed effects, whilst a principal component analysis was used to account for population structure (see below), with the principal components included as a covariate. In the case of the chicken, males have a larger body mass (F_8 birds, t-test $P<9x10^{-88}$), as well as a larger brain mass (F_8 birds, t-test $P<8x10^{-20}$). To account for a

particular QTL varying between the sexes, a sex-interaction effect was added where significant. Two locus (digenic) epistatic analysis was performed as per the guidelines given in the R/qtl handbook [11]. A global model incorporated standard main effects, sex interactions and epistasis was built up starting with the most significant loci and working down for each trait. For brain mass QTL analysis (whole brain and individual regions), body mass was not included as a covariate to prevent QTL overlaps between brain and body mass being removed through for body mass. The exception to this was fitting an additional model with all detected brain mass QTL in conjunction with a body mass covariate to assess the relative impacts of the genetic loci and body mass on brain mass. Sex, batch and the population structure PC were included as covariates for all brain mass QTL analyses, with a sex-interaction term also fitted if significant. Almost all brain regions were correlated with one another (with the exception being no correlation between optic tectum and brainstem mass), therefore no multiple testing correction was needed for mapping multiple phenotypes (see supplementary table 1). Details regarding significance thresholds, family structure and selective sweep clustering analysis are given in the supplementary methods section.

Significance thresholds

Significance thresholds for all QTL analysis were calculated using permutation tests [12,13]. A suggestive significance level of a genome-wide 20% threshold was used (due to this being more conservative than the standard suggestive threshold [14]). The approximate significant threshold was LOD ~4.4, whilst the suggestive threshold was ~3.6 Confidence intervals (C.I.) for each QTL were calculated with a 1.8 LOD drop method (i.e. where the LOD score on either side of the peak decreases by 1.8 LOD) [15]. The nearest marker to this 1.8 LOD decrease was then used to give the C.I. in

megabases. Epistatic interactions were also assessed using permutation thresholds generated using R/qtl, once again with a 20% suggestive and 5% significant genome-wide threshold used (using the guidelines given in [11]).

Family structure

Thresholds and analysis for an advanced intercross can potentially be problematic, as the family structure can lead to non-syntenic association [16], whereby regions that are in LD with the actual QTL will appear significant, resulting in false positive results. To avoid this, we firstly used a large number of families (n=118) to generate the total number of individuals, to break down this sub-structure as much as possible. For example, if only one offspring were used per family, no family structure would exist and the population would function exactly as recombinant inbred lines [16]. A PCA approach was used to control for any residual family structure [17], despite these small family sizes. This was performed by first calculating the ten strongest PCs, then these being tested for significance in each QTL regression. All significant PCs were retained in the final model. This approach allowed us to both control for population substructure and also test for epistatic interactions, a feature that is impossible using other packages designed for advanced intercross QTL analysis.

Selective Sweep Clustering Analysis

The clustering test was performed using a permutation test based on the total length of the chicken genome (1.09Gb), which then had a number of regions equal to the number of each type of QTL detected in the F_8 cross (e.g. whole brain mass QTL, cerebellum QTL) and the number of selective sweeps (n=133) randomly distributed along it. The mass of these regions was equal to the average C.I. of QTL from the

intercross (5Mb) and the average mass of the selective sweeps (40kb), and tested against the observed number of overlaps between the detected QTL and the selective sweeps. This was repeated 1000 times, with the number of overlaps recorded each time used to generate a significance value.

Fecundity Phenotypic Measures

One major behavioural change caused by domestication in chickens is reduced brooding behaviour. In RJF brooding behaviour in females is associated with the cessation of egg laying followed by nesting after a clutch of 6-10 eggs have been laid, but selection for persistent egg production during domestication has resulted in a reduction in the incidence of this behaviour [18] particularly in Mediterranean breeds such as the White Leghorn in which brooding behaviour is rarely observed [19]. Therefore one method for ascertaining if a chicken is brooding is to perform two fecundity trials, one in which the eggs are removed daily, followed by another in which the birds are allowed to retain the eggs laid. The number of eggs laid in the second trial is then deducted from the number of eggs laid in the first trial to calculate a 'brooding index'. The lower this number is the less broody the individual is (with negative values indicating a female laid more eggs during the brooding trial than the fecundity trial). Initially birds were housed individually and eggs were collected daily over a two-week period for the first trial. The second trial was performed immediately after the first and was identical except birds were given two dummy eggs to incubate and were allowed to keep all eggs laid over a ten-day period. Because the brooding trial was four days shorter than the fecundity trial (with the exception of one batch), and to make the brooding indices between the two trials more interpretable, we extrapolated the number of eggs in the second trial to 14 days. We excluded 11

females that laid no eggs in the first trial and 55 that laid no eggs in the second trial. Chickens were reared and tested in five separate batches. In the case of the first two batches, the number of females exceeded the number of individual cages available for testing, resulting in assays being staggered in two sub-batches. This was then included as a covariate in subsequent QTL analyses.

Correlations Between Brain Region Mass And Brooding Behaviour

Correlations were performed using the linear model function in R [7]. Total mass and proportion of total brain mass (i.e. region mass divided by total brain mass) for the cerebellum and cerebral hemispheres were modelled against brooding behaviour. Body mass at slaughter was added as covariate, whilst rearing batch was included as a fixed factor. A total of 123 birds were used in the analysis.

Relative and Total Brain Mass and Brain Region Mass Differences between Domestic and Wild Birds

The ontogenetic comparison of wild Red Junglefowl and domestic White Leghorn birds was performed at each age point (six age points used in total – from weeks one, two, four, ten, fifteen and adult). Eight to seventeen birds from each population (RJF and WL) were used for each time point comparison (1st week: 10-RJF and 10-WL, 2nd week: 10-RJF and 10-WL, 4th week: 10-RJF and 10-WL, 10th week: 10-RJF and 8-WL, 15th week: 10-RJF and 8-WL, Adulthood: 11-RJF and 17-WL), with both absolute and relative mass calculated. A 2-sample t-test was used to compare differences between RJF and WL individuals for absolute brain region mass, using the R statistical software package [7].

SUPPLEMENTARY RESULTS

Brain regions exhibit consistently different mass between domestic and wild birds

By measuring brains from RJF and domestic chickens (WL) from 1-week of age until sexual maturity we show that RJF brain regions weigh about ~85% of the total mass of their domestic counterparts (cerebral hemispheres ~83%, optic tectum ~88%, brainstem ~90%, cerebellum ~81%), with this mass difference being largely consistent throughout post-hatch growth (Supplementary figure 3B-E). The relative mass of the cerebral hemispheres and cerebellum were consistently larger in WL than in RJF throughout post-hatch growth (Supplementary figure 3A and D, while the optic tectum and brainstem regions were consistently proportionally larger in RJF than in WL (see Supplementary figure 3B and C). Each brain region grows continuously from the chick-phase (1-week old) until sexual maturity in domestic and RJF chickens, but the relative mass of each brain region changes (Supplementary figure 3). In general for all birds (regardless of breed), there is a change in the different regions from the chick-phase until sexual maturity. In the case of the cerebral hemispheres, the relative mass changes by around 6% during development, while the relative mass of the brainstem region is essentially fixed and the relative mass of the optic tectum decreases by 3%. The relative mass of the cerebellum increases by 2% during development. The differences between RJF and domesticated (WL) chickens is also generalised to broilers (chickens produced for meat), with broilers at two weeks of age showing similar changes in brain composition as WL (see supplementary figure 4).

SUPPLEMENTARY REFERNCES

1. Jarvis, E. D. *et al.* Avian brains and a new understanding of vertebrate brain evolution. *Nature Reviews Neuroscience* **6**, 151-159 (2005).
2. Balanoff, A. M., Bever, G. S., Rowe, T. B. & Norell, M. A. Evolutionary origins of the avian brain. *Nature* **501**, 93-96 (2013).
3. Striedter, G. F. & Charvet, C. J. Developmental origins of species differences in telencephalon and tectum size: morphometric comparisons between a parakeet (Melopsittacus undulatus) and a quail (Colinus virgianus). *Journal of Comparative Neurology* **507**, 1663-1675 (2008).
4. Wylie, D. R., Gutierrez-Ibanez, C., Pakan, J. M. & Iwaniuk, A. N. The optic tectum of birds: mapping our way to understanding visual processing. *Canadian Journal of Experimental Psychology/Revue canadienne de psychologie expérimentale* **63**, 328 (2009).
5. Reiner, A. *et al.* Revised nomenclature for avian telencephalon and some related brainstem nuclei. *Journal of Comparative Neurology* **473**, 377-414 (2004).
6. Scherle, W. A simple method for volumetry of organs in quantitative stereology. *Mikroskopie* **26**, 57 (1970).
7. R: A language and environment for statistical computing (R Foundation for Statistical Computing, Vienna, Austria, 2012).
8. Sambrook, J., Fritsch, E. F. & Maniatis, T. *Molecular Cloning: A Laboratory Manual*. (Cold Spring Harbour Laboratory Press, 1989).
9. Johnsson, M. *et al.* A Sexual Ornament in Chickens Is Affected by Pleiotropic Alleles at HAO1 and BMP2, Selected during Domestication. *PLoS Genetics* **8**, e1002914, doi:10.1371/journal.pgen.1002914 (2012).

10 Broman, K. W., Wu, H., Sen, S. & Churchill, G. A. R/qtl: QTL maping in experimental crosses. *Bioinformatics* **19**, 889-890 (2003).

11 Broman, K. W. & Sen, S. *A guide to QTL mapping with r/qtl*. (Springer, 2009).

12 Churchill, G. A. & Doerge, R. W. Empirical Threshold values for Quantitative Trait Mapping. *Genetics* **138**, 964-971 (1994).

13 Doerge, R. W. & Churchill, G. A. Permutation Tests for Multiple Loci Affecting a Quantitative Character. *Genetics* **142**, 285-294 (1996).

14 Lander, E. S. & Kruglyak, L. Genetic Dissection of Complex Traits: Guidelines for Interpreting and Reporting Linkage Results. *Nature Genetics* **11**, 241-247 (1995).

15 Manichaikul, A., Dupuis, J., Sen, S. & Broman, K. W. Poor Performance of Bootstrap Confidence Intervals for the Location of a Quantitative Trait Locus. *Genetics* **174**, 481-489, doi:10.1534/genetics.106.061549 (2006).

16 Peirce, J. L. *et al.* Genome Reshuffling for Advanced Intercross Permutation (GRAIP): Simulation and Permutation for Advanced Intercross Population Analysis. *PLoS ONE* **3**, e1977 (2008).

17 Wu, C., DeWan, A., Hoh, J. & Wang, Z. A Comparison of Association Methods Correcting for Population Stratification in Case–Control Studies. *Annals of Human Genetics* **75**, 418-427, doi:10.1111/j.1469-1809.2010.00639.x (2011).

18 Hutt, F. B. *Genetics of the Fowl*. Vol. 274 (McGraw-Hill New York, 1949).

19 Romanov, M., Talbot, R., Wilson, P. & Sharp, P. Genetic control of incubation behavior in the domestic hen. *Poultry science* **81**, 928-931 (2002).

SUPPLEMENTARY FIGURE LEGENDS

Supplementary figure 1. Picture showing a whole chicken brain and a brain dissected into the four regions (Cerebral hemisphere, Optic tectum, Brainstem and Cerebellum).

Supplementary figure 2. Brain mass (g) versus brain volume measures (cm^3) in A) whole brain and B) brain regions (lower graph).

Supplementary figure 3. (A-C) Changes in absolute mass in grams (solid lines) and relative mass (% of total brain mass: dotted lines) of A) Cerebral hemisphere, B) Optic tectum, C) Brainstem, and D) Cerebellum, in White leghorn (black lines) and Red Junglefowls (red lines) from 1-week of age until adulthood. For (A-D) T-test comparisons between WL and RJF brain region absolute mass values were made within each time point, with * indicating $P<0.05$, ** indicating $P<0.01$.

Supplementary figure 4. Relative mass (Mean +/- s.e.) of each of the four brain regions (Cerebral hemisphere, Optic tectum, Brainstem and Cerebellum) in Red Junglefowl (RJF), White leghorn (WL) and Broilers (B).

SUPPLEMENTARY TABLE LEGENDS

Supplementary table 1. (A) Phenotypic correlations between brain regions using absolute mass values (B) Phenotypic correlations between brain regions using relative mass values. Correlations given as Pearson correlation statistic, with * indicating significance at $P<0.05$, ** indicating significance at $P<0.01$, and *** indicating significance at $P<0.001$. Tables are symmetrical, therefore duplicate values are not filled in.

Supplementary table 2. QTL information for all QTL. Includes locations (both the chromosome and the position in centiMorgans), % variance explained by each QTL

(r-squared), additive and dominance effect sizes (positive values for additive values indicate a larger QTL effect size in domestic genotype birds, negative a larger value in wild genotype birds). The lower and upper bounds of the 95% confidence interval (C.I) are noted. The total QTL region is therefore the region bounded between these two limits. Locations of selective sweeps are also provided, with AD indicating the sweep is present in both Broiler and Layer birds, and LR indicating the sweep is specific to Layer birds. For sweeps present in cerebellum and total brain mass QTL any genes present within sweeps are also provided after the sweep location. Cerebellum QTL are marked in bold.

Supplementary table 3. Covariates and interactions associated with detected QTL, ordered by chromosome.

Supplementary table 4. DXA measures of lean and fat mass in domestic and RJF chickens.

241 **Supplementary figure 1**

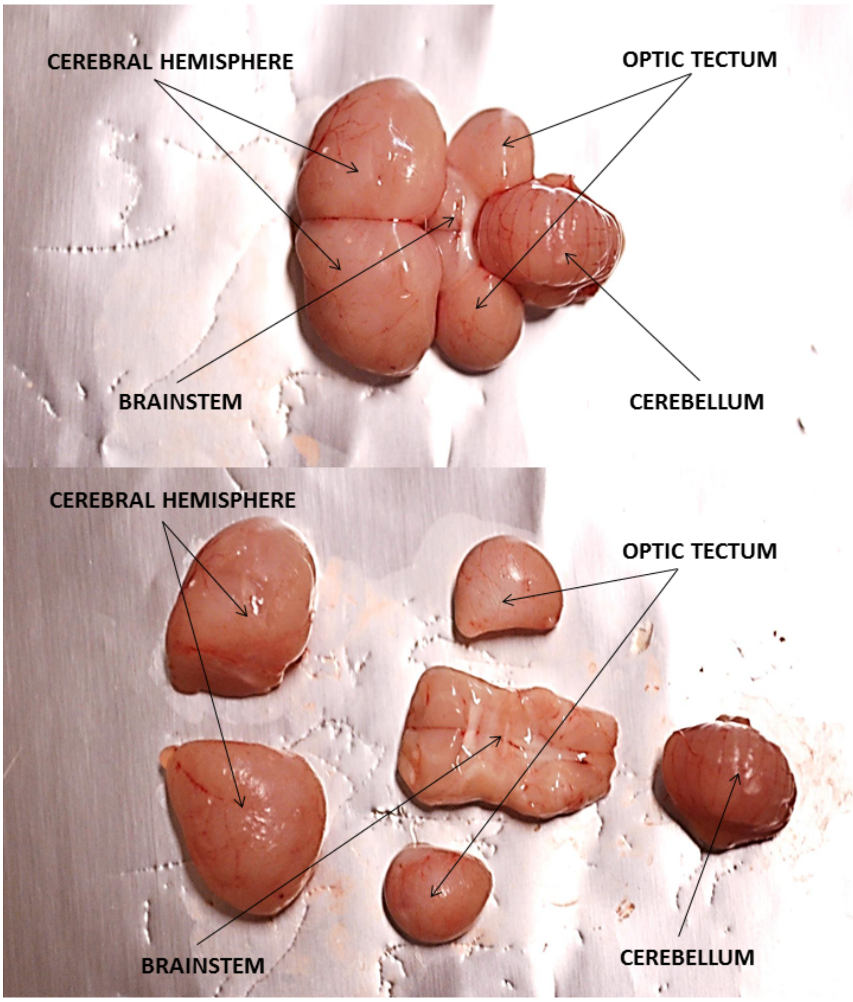

Supplementary figure 2

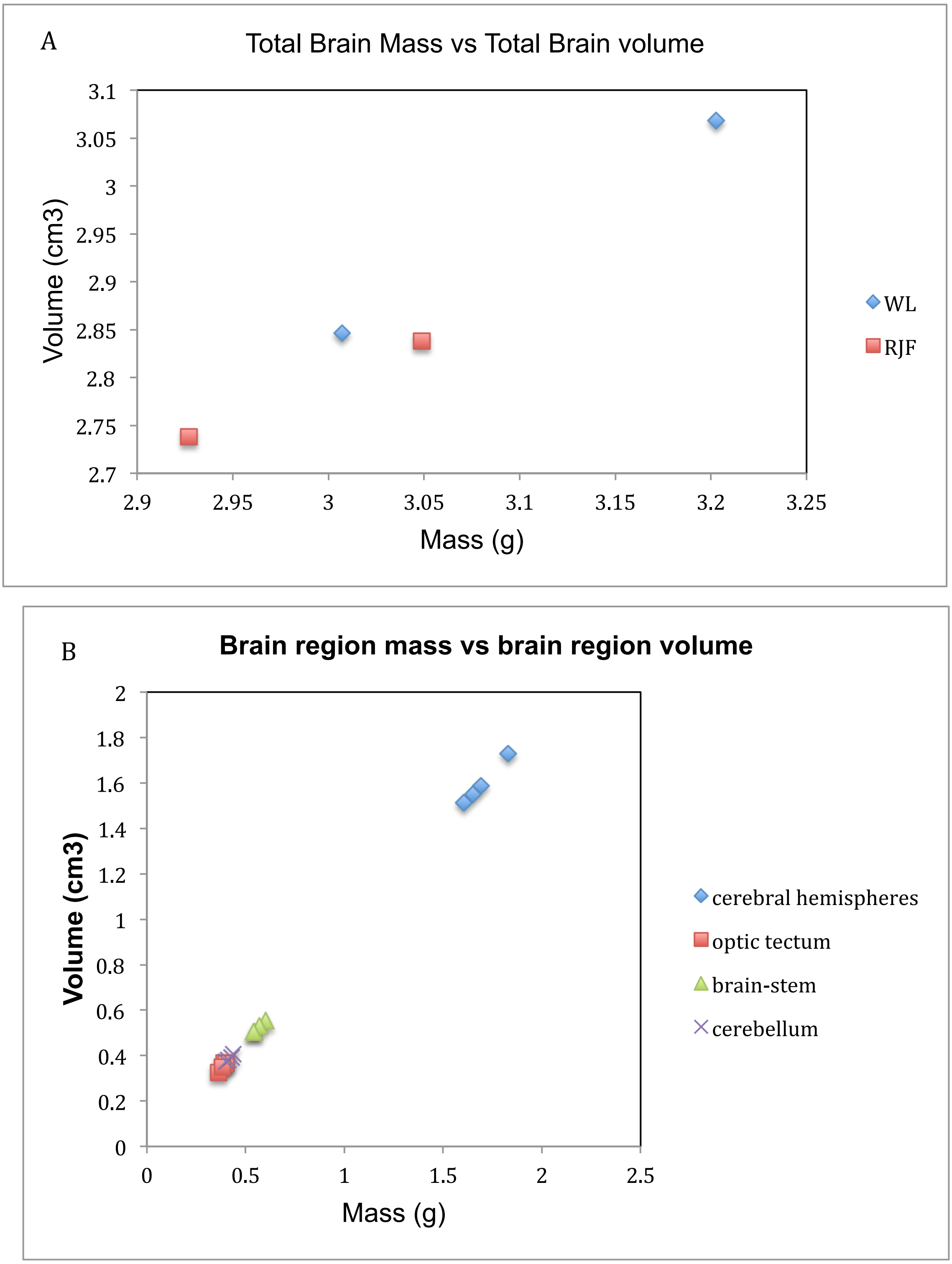

244 Supplementary figure 3

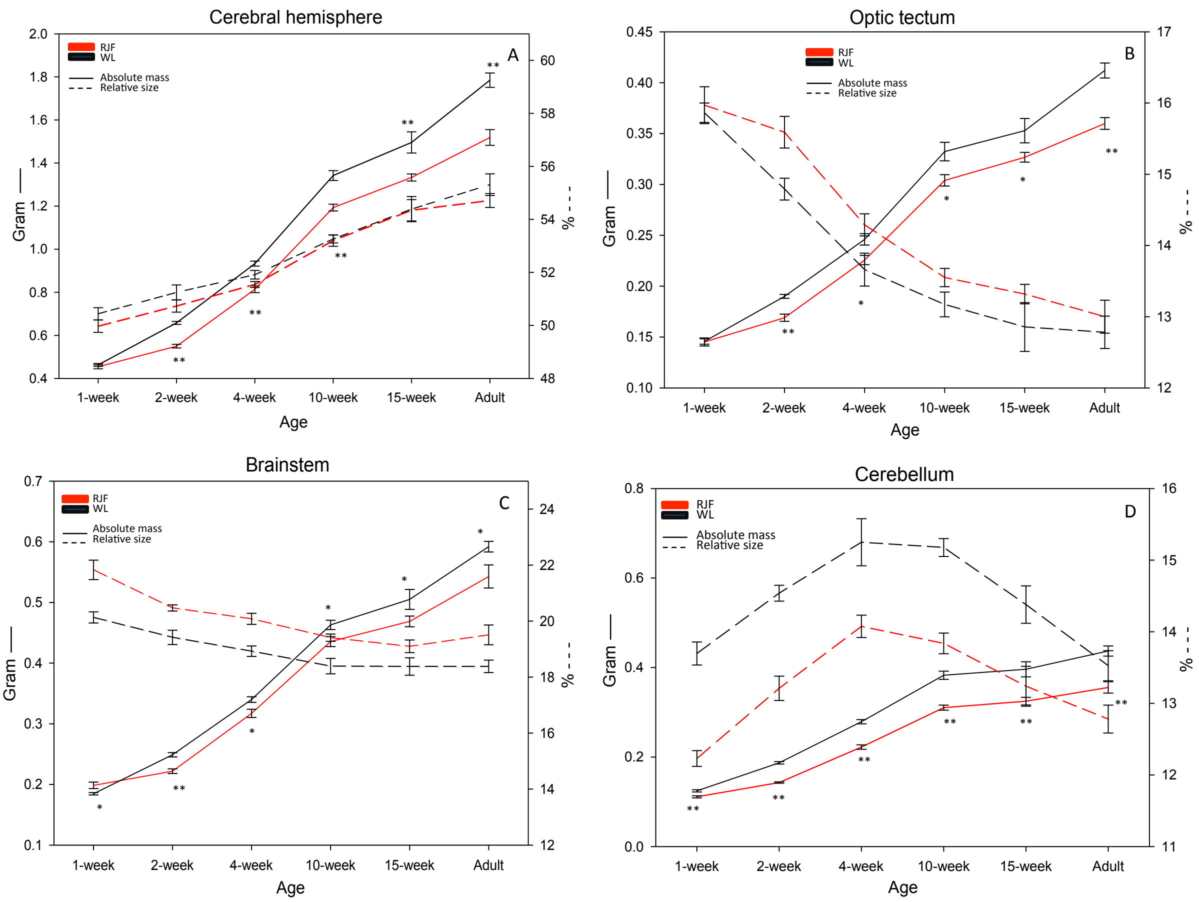

245 **Supplementary figure 4**

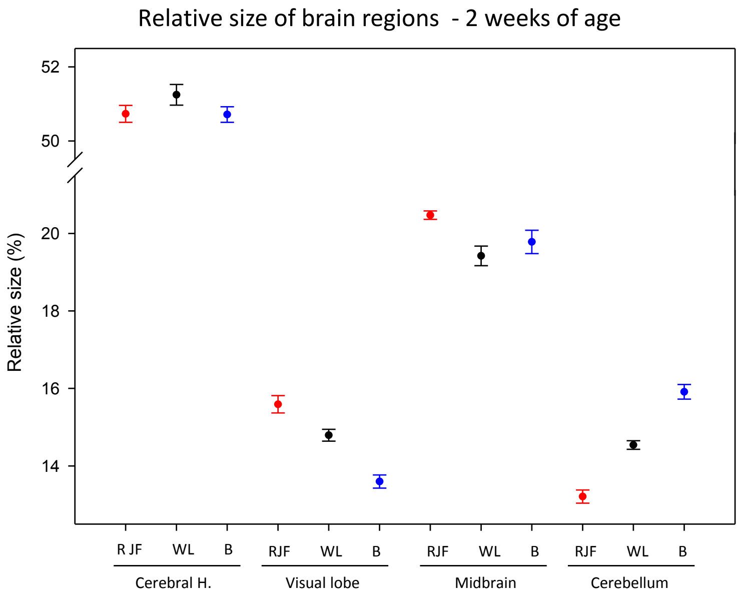

Supplementary table 1. (A) Phenotypic correlations between brain regions using absolute mass values. (B) Phenotypic correlations between brain regions using relative mass values. Correlations given as Pearson correlation statistic, with * indicating significance at P<0.05, ** indicating significance at P<0.01, and *** indicating significance at P<0.001.

**A**			
**Brain region mass**	Optic tectum	Brain stem	Cerebellum
Cerebral hemispheres	0.39***	0.27***	0.48***
Optic tectum	-	-0.06	0.22***
Brainstem	-	-	0.28***
Cerebellum	-	-	-
**B**			
**Brain region relative mass**	% Optic tectum	% Brain stem	% Cerebellum
% Cerebral hemispheres	0.07	-0.70***	-0.36***
% Optic tectum	-	-0.51***	-0.16**
% Brainstem	-	-	-0.18**
% Cerebellum	-	-	-

Supplementary table 2. QTL information for all QTL. Includes locations (both the chromosome and the position in centiMorgans), % variance explained by each QTL (r-squared), additive and dominance effect sizes (positive values for additive values indicate a larger QTL effect size in domestic genotype birds, negative a larger value in wild genotype birds). The lower and upper bounds of the 95% confidence interval (C.I) are noted. The total QTL region is therefore the region bounded between these two limits. Locations of selective sweeps are also provided, with AD indicating the sweep is present in both Broiler and Layer birds, and LR indicating the sweep is specific to Layer birds. For sweeps present in cerebellum and total brain mass QTL any genes present within sweeps are also provided after the sweep location. Cerebellum QTL are marked in bold.

trait	chr	pos	LOD	r-sq	add +/- s.e	dom +/- s.e.	lower CI	upper CI	selective sweeps present
body mass (212 days)	1	510	43,5	21,5	235 +/- 28	-20 +/- 38	507	516	
relative cerebral hemisphere	1	596,2	7,7	8,6	-0.004 +/- 0.002	0.001 +/- 0.002	593	607	43.7 AD, 44.9 AD
total cerebral hemisphere mass	1	1078	5,7	4,2	0.04 +/- 0.008	-0.003 +/- 0.01	1058	1084	
relative cerebral hemisphere	1	1221	11,3	13,1	0.004 +/- 0.006	-0.03 +/- 0.007	1212	1225	
total brain mass	1	1516	5,3	3,3	0.06 +/- 0.02	-0.06 +/- 0.04	1494	1583	119.46 LR (*SPAC17A2*), 127.88 LR (*ARHGAP6*)
**total Cerebellum mass**	1	1593	13,5	9,9	0.012 +/- 0.005	-0.023 +/- 0.007	1586	1598	127.88 LR
**relative Cerebellum**	1	1945	4,8	4,8	0.003 +/- 0.001	0.005 +/- 0.002	1931	1956	179.66 LR (*UBL3*), 182.6 LR (*FGF9*)
**total Cerebellum mass**	1	2204	9,3	6,6	0.0004 +/- 0.004	-0.004 +/- 0.005	2196	2224	*MAP6, CCKBR, PLEKHB1*
relative brainstem	3	403	5,4	7,4	-0.052 +/- 0.011	0.06 +/- 0.01	386	407.93	
**total Cerebellum mass**	3	448	8,5	6	0.005 +/- 0.005	0.022 +/- 0.007	442	458	62,62 LR (*KNF217*), 64.04 LR (*SERINC1*)
total brain mass	3	448	8,7	5,6	-0.12 +/- 0.07	0.25 +/- 0.09	444	454	62,62 LR (*KNF217*), 64.04 LR (*SERINC1*)
brooding	4	154	9	12.5	-3.74 +/- 0.83	3.25 +/- 1.42	150	163	
body mass (212 days)	4	265	8,6	4,7	8.1 +/- 9.8	19.8 +/- 12.3	254	274	
brooding	4	492	7,8	10.6	1.16 +/- 0.36	-0.65 +/- 0.48	470	502	72.46 AD. 76.14 AD. 78.42 LR. 80.32 AD. 80.38 AD. 80.44 AD. 80.76 AD
relative optic tectum	4	205.7	4,7	6,3	0.005 +/- 0.005	0.016 +/- 0.007	201	223	28.02 AD, 29.5 AD
**relative Cerebellum**	5	124	4,8	4,8	0.002 +/- 0.004	-0.03 +/- 0.006	106	144	18.8 LR (*FGF3*), 19.4 LR (*SHANK1*), 20.2 LR (*CAT*), 20.5 LR (*CD44*)
body mass (212 days)	6	207	5	1,9	26 +/- 8	30 +/- 11	195	214	
**relative Cerebellum**	7	159	7,4	7,6	0.005 +/- 0.002	0.004 +/- 0.002	150	171	
**total Cerebellum mass**	7	174	5,9	4,1	0.019 +/- 0.005	0.003 +/- 0.005	150	176	23.04 AD (*TANK1*)
total brain mass	7	200	6,6	4,2	0.02 +/- 0.018	-0.04 +/- 0.03	190	212	23.04 AD (*TANK1*), 23.1 AD, 25.42 LR (*BIN1*), 25.9 LR
total cerebral hemisphere mass	8	51	4,8	3,5	0.05 +/- 0.01	-0.01 +/- 0.02	36	87	8.98 AD
brooding	9	0	5,1	6.6	0.09 +/- 0.33	0.03 +/- 0.47	0	16	
total brain mass	9	51.1	13,6	8,9	0.018 +/- 0.017	-0.04 +/- 0.02	48	60	
total cerebral hemisphere mass	10	249	5,9	4,3	-0.07 +/- 0.01	-0.05 +/- 0.02	232	257	19.3 LR
body mass (212 days)	12	64	4,4	1,7	35 +/- 10	20 +/- 14	45	79	
total cerebral hemisphere mass	12	232	5,3	3,8	-0.04 +/- 0.008	-0.03 +/- 0.01	218	240	17.2 AD
brooding	13	54	10	14.0	-4.39 +/- 1.62	4.83 +/- 2.84	44	80	3.68 LR
relative cerebral hemisphere	21	8	3,5	3,8	-0.008 +/- 0.002	-0.003 +/- 0.003	0	19	0.1 AD
body mass (212 days)	24	14	9,5	5	0.8 +/- 9.1	38 +/- 11	6	18	
body mass (212 days)	27	68	4,2	1,6	38 +/- 11	-1.8 +/- 9.8	56	80	

Supplementary table 3. Covariates and interactions associated with detected QTL, ordered by chromosome.

Trait	covariates	Interactions
body mass (212 days)	sex, batch, PCs	
relative cerebral hemisphere mass	sex, batch, PCs,	1@596.2:1@1221.0
total cerebral hemisphere mass	sex, batch, PCs,	
relative cerebral hemisphere mass	sex, batch, PCs,	1@596.2:1@1221.0, 1@1221.0:sex
total brain mass	sex, batch, PCs,	1@1516.0:9@51.1
total Cerebellum mass	sex, batch, PCs,	3@448:7@174, 1@1593:1@2204
relative Cerebellum mass	sex, batch, PCs	
total Cerebellum mass	sex, batch, PCs,	1@1593:1@2204
relative brainstem mass	sex, batch, PCs	sex:3@403.0
total Cerebellum mass	sex, batch, PCs,	3@448:7@174
total brain mass	sex, batch, PCs,	3@448.0:sex
Brooding	w212, batch, part_fec, PCs	4@154.0:13@54.0
body mass (212 days)	sex, batch, PCs	
Brooding	w212, batch, part_fec, PCs	4@492.0:9@0.0
relative optic tectum mass	sex, batch, PCs	Sex
relative Cerebellum mass	sex, batch, PCs	sex:5@124.0
body mass (212 days)	sex, batch, PCs	
relative Cerebellum mass	sex, batch, PCs	
total Cerebellum mass	sex, batch, PCs,	
total brain mass	sex, batch, PCs,	9@51.1:7@200.0
total cerebral hemisphere mass	sex, batch, PCs,	
Brooding	w212, batch, part_fec, PCs	4@492.0:9@0.0
total brain mass	sex, batch, PCs,	1@1516.0:9@51.1, 9@51.1:7@200.0
total cerebral hemisphere mass	sex, batch, PCs,	
body mass (212 days)	sex, batch, PCs	
total cerebral hemisphere mass	sex, batch, PCs,	
Brooding	w212, batch, part_fec, PCs	4@154.0:13@54.0
relative cerebral hemisphere mass	sex, batch, PCs,	
body mass (212 days)	sex, batch, PCs	
body mass (212 days)	sex, batch, PCs	

Supplementary table 4. Dual-energy x-ray absorptiometry (DXA) measures of lean and fat mass in domestic and Red Junglefowl (RJF) chickens.

trait	WL females	RJF females	WL males	RJF males
total mass (g)	1408	818	1566	1221
lean mass (g)	910	684	1349	944
fat mass (g)	427	98	146	221
% lean	0.65	0.84	0.86	0.77
% fat	0.30	0.12	0.09	0.18
DXA Bone Mineral Density	467	331	389	376
DXA Bone Mineral Content	72	35	71	56
BMD/ mass	0.33	0.40	0.25	0.31
BMC/ mass	0.05	0.04	0.05	0.05
